# Sicilian Black Pig: An Overview

**DOI:** 10.3390/ani10122326

**Published:** 2020-12-07

**Authors:** Alessandro Zumbo, Anna Maria Sutera, Giuseppe Tardiolo, Enrico D’Alessandro

**Affiliations:** Unit of Animal Production, Department of Veterinary Sciences, Via Palatucci snc, Messina University, 98168 Messina, Italy; alessandro.zumbo@unime.it (A.Z.); asutera@unime.it (A.M.S.); gtardiolo@unime.it (G.T.)

**Keywords:** Sicilian black pig, autochthonous Italian breed, phenotype traits, productive traits, rearing systems, food productions, genetic traits, biodiversity

## Abstract

**Simple Summary:**

The conservation of the genetic variability of animals used for food production and non-food raw materials and services is a problem of primary importance at a global level. In recent years, conservation of biodiversity in livestock species has been favoring the need to preserve genetic variability of the autochthonous breeds, exploiting them in the context of production systems. In this context, a precious genetic reserve is represented by autochthonous breeds used for the production of typical products used in Italian gastronomic traditions, of which some organoleptic properties of their meats that could disappear due to severe selection programs are being recovered. Currently, the survival of autochthonous breeds is linked to various reasons such as their rusticity, i.e., the adaptability to difficult environmental conditions, and to the higher market value of their productions obtained according to traditional methods compared to the industrial production types. As information on autochthonous Italian pigs is limited, further research aims at making better use of these breeds and at increasing the knowledge of their genetic variability.

**Abstract:**

The Sicilian black pig (SB) (Nero Siciliano), also known as the Nero dei Nebrodi, Nero delle Madonie, or Nero dell’Etna pig ecotype, is an autochthonous Italian breed. The origins of this breed date back to Greek and Carthaginian dominations. In ancient times, its breeding was fairly common throughout Sicily, registering only a temporary reduction during the Arab domination. This breed is known primarily for its distinctive black coat, although some individuals display wattles and a partially or wholly white face. The SB pig has a birth rate with an average per sow of 7.6 piglets, each of 1.4 kg live body weight, showing an average daily gain (ADG) of 346 g/day during the fattening period. Slaughter generally takes place at an average age of 390 days, with an average live weight of 95 kg. This breed also appears to withstand adverse climatic conditions and resist disease. The purpose of this manuscript is to offer a general overview regarding the Sicilian Black pig and to consider the recent findings related to genome investigation. The recent application of Next Generation Sequencing (NGS) technologies in the study of the genome of autochthonous breeds showed that polymorphisms of some candidate genes for production performance and phenotypic traits represent important information for selection processes. The protection of autochthonous breeds, intended as sources of genomic diversity for the further improvements of pigs for commercial use, constitutes a valuable opportunity to create new sustainable pig chains.

## 1. Introduction

The Nero Siciliano pig (Sicilian black (SB) pig) is an autochthonous Italian breed from Sicily [1], a Mediterranean island in southern Italy. Historical traces, such as fossil remains and written texts, reveal its presence since the Greek and Carthaginian periods (seventh–sixth century BC). According to Chicoli (1870) [2], even the Greek poet Omero in his stories mentioned the existence of a black pig that was highly rustic, almost wild, and completely free in the woods. The breeding of black pigs in Sicily was known in Rome as early as the second century BC. In the ninth century BC, pig farming experienced a temporary reduction during the Arab colonization in Sicily for religious reasons. However, this breeding was recovered with the Norman conquest [2]. The origin of the SB pig is controversial. The well-defined characteristics suggest that this breed descends from various breeds and pig populations deriving from the Neapolitan black-haired breed [3]. The SB breed is considered as an expression of ethnic–genetic heterogeneity, showing an evident polymorphism influenced by the natural living environment, the rearing systems, and the type of targeted production system. According to Pino [3], the breed variants identified in general terms are to be referred to as Casertana (Pelatella), Cinta Senese, and Parmigiana for Italians, as the Large Black, Large White, and Berkshire for the English, and as Chester White and Poland China for Americans. Among the mentioned breeds, the Casertana is the most ancient, even though, for commercial reasons and low productivity, its breeding registered an attenuation [3]. Further authors have conducted studies on the above-mentioned breeds. Besides the Large Black and Large White reproducers, the Sicilian Livestock Research Institute imported subjects of the Casertana breed characterized by low fertility and affinity with the SB pig [4]. For example, Marchi [5] studied the influence of the Neapolitan breed and Cassella [6] on Calascibetta pigs bred in the province of Enna. Cassella [6] suggested that SB pigs, such as the North African one, could have origins from the Neapolitan breed. The Large Black has also been present in Sicily, directly introduced into the island from England. This pig is known to be a good grazer, characterized by very large, long, and drooping ears, which could limit its visibility in open spaces, thus conditioning the possibility of running openly in the pastures [4]. The Large White breed was introduced in Italy by Zanelli and later in Sicily by Tucci [6]. This breed was used alone or crossed with other breeds even if it showed weak performances in an extensive condition because of the absence of rusticity [6]. The SB breed is generally considered an “Indigenous Sicilian” population, which for some authors derives from an unique autochthonous line [6,7], for others from individuals of the “Neapolitan” breed [8], and for others from the “Iberian type of Sanson” [2,9,10]. According to Chicoli (1870) [2], the Iberian type of Sanson was based on some typical differential characteristics of the skeleton (mainly the number of dorsal and lumbar vertebrae), defining a brachycephalic morphological type (Asian pig) and a dolichocephalic type (pigs from southern Europe), the latter of which describes SB pigs. Chicoli described various pig breeds in Sicily that lead to the “Neapolitan” breed with a characteristic “ordinary” black coat. These breeds have been listed as follows: (i) Saint Agata di Militello; (ii) Castelbuono; (iii) Trapani; (iv) Patornese; (v) and Cesarotana. According to Romolotti [11], the SB pig was the only one with well-defined characteristics. This could be in line with what was reported by Marchi and Pucci [12] that the Mediterranean areas would be influenced by the introduction of foreign blood, corroborating the fact that the better-defined breeds would have been observed in the island. Furthermore, breeding in the thickest woods in the wildest regions of the island could play as a barrier in countering possible genetic pollution.

## 2. Morphological Characteristics

**Figure 1:**
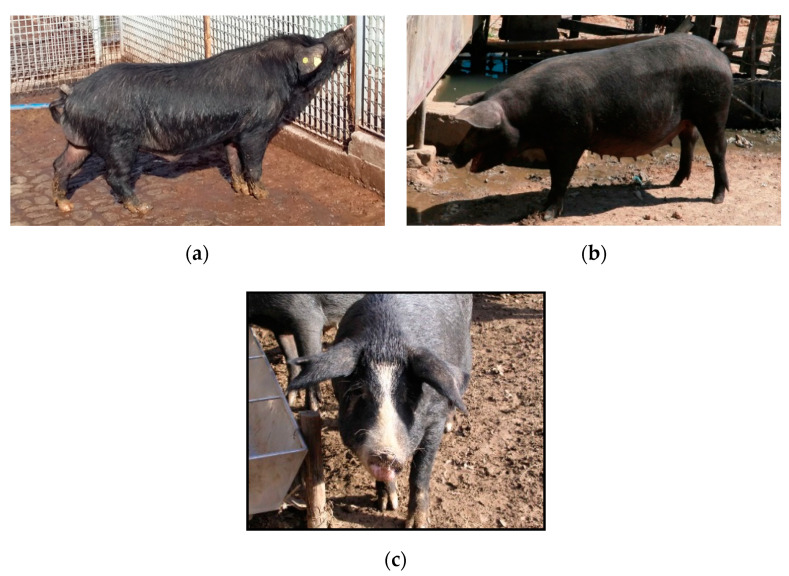
Pigs with different coat colour patterns: Sicilian black sow (**a**), Sicilian black boar (**b**), Facciolo pig (**c**).

These animals are characterized by a black coat with very thick black slate skin that features coarse black hairs, as well as course black hairs on the cervical that create a mane. Some of them are called “Faccioli pigs” due to their partially or totally white face. Its head shows a remarkable development, a long and straight profile, and a narrow and inclined muzzle, while the nasal profile of the forehead shows a tendency to be straight, sometimes presenting *Keloidism* [3]. The ears are small and obliquely turned upwards, with the tips carried horizontally forward [13]. The limbs are of medium length and sturdy and present dry joints and strong nails. The height at the withers is approximately 62–67 cm (see Figure 1). SB pigs are known to be adapted to difficult conditions and are valued for their reproductive performance, disease resistance, and meat production [14,15,16,17].

In Table 1, the information relating to adult body morphology of the SB pig breed (ANAS, [13]) expressed as an average value is shown. Body length shown refers to measurement.

## 3. Monitoring and Protection of Breed

In 2001, the SB pig breed received official recognition as a native breed, with a population of ~13,200, ~4000 of which were sows and ~1500 of which were boars, from over 115 farms (ANAS—Italian Pig Breeders Association, 2020). In 2003, a consortium called “Consorzio di Tutela Suino Nero dei Nebrodi”, supported by the Regional Breeders Association of Sicily in the province of Messina, was established. In 2005, an official demand to recognize fresh Sicilian black meat with the Protected Denomination of Origin (PDO) was issued to Ministry of Agricultural, Food and Forestry Policies. An official PDO request was also initiated for Nero Siciliano’s cured ham in 2011.

## 4. “Plein Air” and Productive Performances

The importance of the SB pig as an autochthonous genetic type (AGT) is well-known, thus representing a cultural and historic heritage. To overcome the feeding uncertainty connected with the climate and season, common feeds such as grains, with some vitamins and minerals as well as suitable shelters to serve the animals according to their necessity and autonomously, were made available. These expedients created the conditions to carry out the so-called “élevage en plein air” [16,17]. The “plein air” in fact can be considered an evolution of the extensive rearing system, as it provides for the confinement of animals in fenced areas, the presence of water troughs, shelters, and food supply systems. To evaluate the quantitative and qualitative responses, animals reared in extensive conditions and those “en plein air” were compared, determining performance “in vitam” and “post mortem” [18]. Observations from summer to autumn showed an average daily gain (ADG) of 77 g for the animals reared in extensive conditions, while animals raised “en plein air” showed an ADG of 320 g [19]. In pigs reared in extensive systems, the non-esterified fatty acids (NEFAs) associated with fat catabolism had significantly higher values during summer months, during which the animals lost fat up to a physiological balance of 0 ADG, indicating the high energetic requirements. In autumn, these parameters were low [19,20]. The notable increase in glucose in the months of July and August could be related to stressful situations. In pigs reared “en plein air”, the best results were those relating to the values of energy and protein metabolism [21,22]. The rearing of animals in extensive conditions showed a slaughter weight of 70.88 kg at 250 days of age, while those raised “en plein air” showed a slaughter weight of 79.71 kg at 160 days of age [20]. Pigs reared “en plein air” showed better slaughter yields and back fat thickness (BFT) than pigs reared under extensive conditions, similar to those obtained for different growth performances [23]. Animals reared “en plein air” showed slightly higher biometric data regarding the length of the half carcass and the thoracic depth [24,25].

## 5. Reproductive Characteristics

The reproductive performances are in agreement with those previously described by Matassino and Grasso [17]. However, these reproductive aspects could be influenced by farmer management. The first farrowing for sows occurs around 30 months of age, while their slaughter occurs around 47 months [1]. The relatively young age at which animals are slaughtered could depend on the presence of both sows slaughtered after the first event, generally due to their poor maternal aptitude (cannibalism, crushing piglets, and non-weaning piglets), or sows discarded from the reproductive career. It is actually quite common for the breeder to test the females, keeping only a few for the breeding career. SB sows give birth on average to 1.1 litters per year [26] made up of 6.2 ± 1.71–9 ± 1.98 piglets out of 180 births [27,28], presenting about 1.4 kg live body weight at birth [29,30]. The percentage of stillborn piglets (about 0.4% and 4.8%) and their mortality rate until weaning (about 1.3% and 8.9%) are relatively low. However, in extensive systems, the interval between calving is extended to 332 days [27]. Generally, births occur in wooded areas, where breeders prepare “farrowing cages”, called “zimme”, suitable for nest-building and births.

## 6. Productive Characteristics

The correct growth of a pig and its productive performance can be influenced by the feed type and energetic and protein level of the diet given, in relation to genetic aptitude [31]. Considering the differences in studies concerning the range of live weight covered, the stages for growth performance are reported below as, respectively, initial and average stages of fattening, estimated between about 30 and 60 kg and between 60 and 100 kg of live weight. For SB pigs, the assessment of growth potential under ad libitum feeding conditions indicated that the maximum growth rate was 540 g/day overall in the fattening stage (from 42 to 93 kg live weight) [32]. Assessment of growth potential is limited due to limited information regarding feed intake and the nutritional value of the feed. During the intermediate fattening phase, the average daily feed intake was approximately 1.5–2.2 kg/day, while in the fattening phase overall, this value was approximately 1.7–2.9 kg/day [33,34]. Table 2 summarizes the data available in the literature regarding the growth performance for SB pigs. In Table 2, the ADG is expressed in g, and the values for ADG in fattening periods are reported for the initial and central fattening phases estimated, respectively, between 30 and 60 kg and between 60 and 100 kg.

Other important characteristics of SB pigs are body composition and features of the carcass. Studies available in the literature and considered in this manuscript indicate that the age at slaughter for the SB breed ranges from 169 to 730 days of age [32,33,34,35,36], with a live body weight from 62 to 121 kg [37,38,39,40,41]. According to the SEUROP classification (classification of carcasses defined by the European Union) [42], the yield of dressing was about 80%, and lean meat content ranged from 39.7% to 59.0% [38,39,40,41]. In addition, the BFT value measured was 52 mm at the withers, from 17 to 49 mm at the last rib, and from 30 to 49 mm above the *gluteus medius* muscle [32,33,35,40]. In the studies cited above, no data relating to muscularity measurements were found. Table 3 shows the data concerning body composition and the characteristics of the carcass. Furthermore, the S values for the BFT were measured according to Fat-o-Meter (FOM method) (above the *gluteus medius* muscle and expressed in mm).

## 7. Meat Quality

SB pigs are known for the organoleptic qualities of their meat, such as various flavors, acidity, salinity, and above all a good lipid profile in terms of the ratio of saturated to unsaturated fatty acids [35,36,37,38,39]. Studies conducted on the meat quality indicated an average pH of 6.24 [37,38,39] and 5.58, measured in the longissimus muscle at 45 min and 24 h post-mortem, respectively (see Table 4 for pH 45 and pH 24). The amount of intramuscular fat (IMF) ranged from 2.7% to 10.0%, increasing with the weight of the slaughter. The meat colour shows the following values: 46–61 for L*, 10.1–15.7 for a*, and 4.6–13.4 for b* (see Table 4). The CIELAB colour space (also known as CIE L*a*b*) is described by the Commission Internationale de l’Eclairage (CIE) [35,36,39,41] and expresses colour as three values: L* for the lightness from black (0) to white (100), a* from green (−) to red (+), and b* from blue (−) to yellow (+). Regarding the lipidic profile, in the studies considered, SB had a content of saturated fatty acid (SFA), monounsaturated fatty acid (MUFA), and polyunsaturated fatty acid (PUFA) in the intramuscular fat (IMF) in the longissimus muscle, respectively, of 37.5%, 54.2%, and 8.3% [35,36,41]. The relative amounts of SFA, MUFA, and PUFA in the back fat tissue were 39.0%, 49.4%, and 11.7%, respectively [42,43,44,45]. Another study of the analysis of the fatty acid profile of meat revealed the presence of important fatty acids, such as oleic acid (C18:1n9) at 43.8–45.7%, palmitic acid (C16:0) at 21.7–23.9%, stearic acid (C18:0) at 11.4–3.7%, and linoleic acid (C18:2n6) at 8.46–9.98% [46]. Table 4 summarizes some of the most general meat and fat quality traits evaluated in the longissimus muscle.

## 8. Typical Local Products

Food supplementation is foreseen in all physiological phases, in particular during the finishing phase. The diets are predominantly comprised of acorns, which are very rich in oleic acid. However, the period in the wood could be influenced by the climatic conditions and therefore limited to different seasons, affecting the production and quality of the meat. The products derived from Sicilian pigs was also known in Rome BC [47], and the type salami of Saint Angelo di Brolo (a town in the Messina hinterland) is considered the oldest Italian sausage prepared with SB meat in the Mediterranean region. This statement should not come as a surprise since the earliest representation of salami was probably found in Thebes in 1166 BC [47]. The reputation of SB products is the result of the type of preparation and the particular environment and ecosystem of the territory. The traditional salami of Saint Angelo is known for its ideal organoleptic characteristics in terms of its meat–fat ratio, its tenderness, and the color of the meat, the grain, and the structure of the slices. Furthermore, the aromatic flavor and the color of the fat are satisfactory [44]. A similarity in the chemical composition of the fat and protein is observed between cured ham from SB pigs and cured ham from Parma. In the fatty acid profile, the most represented are palmitic acid, stearic acid, oleic acid, and linoleic acid, comparable to those identified in cured Iberian ham and Parma ham [48,49], with some small differences [50]. In SB ham, a lower SFA content is observed than in Parma ham. The Sicilian product showed similar levels for the MUFA and higher levels for the PUFA, thus indicating more valuable nutritional characteristics [51]. The unsaturated/saturated ratio was similar compared to the Iberian ham and superior to the Parma ham [52]. The main salamis are the Salame Sant’Angelo, which in 2008 gained the status of Protected Geographical Indication (PGI), “cured ham”, “capocollo”, “guanciale”, “sausage”, “lardo”, and “coppa” [52,53].

## 9. Genetic and Genomic Characterization

Genetic research on SB has found variability in candidate genes associated with relevant characteristics of production traits [54]. Sarcolipin (SLN) [55] and ATPase Na+/K+ transporting subunit alpha 2 (ATP1A2) [14] have an allele associated with a greater weight of lean cuts, and they can be considered genes responsible for the growth and adiposity of the carcass. In this context, another gene, cystatin B (CSTB), appears to be strongly linked to average weight gain. The CTSB locus (cathepsin B), mapped on SSC2, allowed for the highlighting of allelic frequencies similar to those observed in the Pietrain breed [55,56] (confirmed presence in the history of the SB pig) with a prevalence of allele 2, (G > C), for which an association with BFT has been hypothesized [14,56]. In the SB pig, two alleles of the ESR locus (estrogen receptor) were identified. Its presence in the SB is to be considered significant, given the low prolificacy observed for this pig and the prospects of improvement in reproductive performance [57,58]. In the cyclic redundancy check locus (halothane sensitivity) (CRC) of SB pigs, the presence of “Nn” genotypes was observed, in addition to the prevalence of the “N” allele. In several subjects, the “n” allele was also involved in halothane sensitivity, linked to malignant hyperthermia (MH) and to PSE defects (Pale, Soft, Exudative) [58]. The “n” allele may have been introduced in SB pigs through the crossbreeding with other breeds [58]. Mutations of the MC1R gene affect the coat color of the pig breeds described. Some of them are breed-specific, thus representing a significant perspective for recognition procedures [59,60]. In SB pigs, a study was carried out on the melanocortin 1 receptor (MC1R) gene [59], in order to use this specific locus in the traceability of the breed and its associated food production. The four allelic forms (ED^1^, ED^2^, E^p^, and e) indicated in the MC1R locus have aroused the interest of the scientific community. The data obtained from these studies highlighted the presence of a considerable genetic diversity in the SB breed, a significant aspect to consider in target selection processes. Furthermore, these data confirm the crossing of SB pigs with breeds such as Hampshire, Landrace, and Pietrain [61]. These breeds are used by breeders with the aim of obtaining more immediate results without considering the possible genetic pollution that could induce the onset of a set of negative characters. The alleles responsible for the black colour of the coat are more frequent. However, the possible function of other genes responsible for the presence of white coat parts in the SB pig is not entirely excluded in the breed standard [61,62,63]. The development of Single-Nucleotide Polymorphism (SNP) panels has allowed for the identification of highly differentiated genomic regions increasing the potency of genome-wide association studies (GWASs) [64]. SNPs represent the primary functional basis of genetic variability, which is reflected in phenotypic differences between and within breeds. In the event that the polymorphisms identified represent exclusive selective footprints of the breed under consideration, these markers can be effectively applied to identify the most favorable genetic variants for the improvement of quantitative traits of zootechnical interest, or in the identification and traceability of products [63,64]. Muñoz et al. [65] analyzed the genomic diversity of 20 European autochthonous pig breeds, including SB, using high-density SNP chips. Among the populations studied, SB showed one of the highest minor allele frequency (MAF) values (0.272) and observed heterozygosity (0.34). Furthermore, this breed showed the lowest linkage disequilibrium (r^2^ = 0.305) considering a short distance (0.00–0.02 Mb). These results are in agreement with the SB pig breeding system, as no selection was made for this breed. Due to the development and application of high-throughput technologies and the reference assembly of the whole sequenced pig genome (Sscrofa11.1), it is now possible to use next generation sequencing (NGS) platforms to further investigate genetic diversity in many breeds and populations [66,67]. To explore the genetic variability of the SB pig, whole genome sequencing and the discovery of variants in SB pigs compared to the last reference genome of the Sus scrofa11.1 were presented. A total of 11 million variants were detected. Of these, ~82% were SNPs, while ~12% and ~5% were short insertions and deletions, respectively [15]. Furthermore, more than 58% of detected SNPs (6,555,556 variants) were heterozygous, while the remaining 42% were found in an alternative homozygosity state. The same study evaluated the sequence diversity in 21 genes in relation to environmental adaptation and reproductive traits selected based on their biological function and/or their proximity to important Quantitative Trait Loci (QTLs) considering local pig breeds. In detail, a total of 6747 variants were identified. Among these, 1132 were new to the dbSNP151 database, consisting of 476 that were heterozygous and 656 in an alternative homozygosity form [15]. Several GWASs have been performed in pigs to identify the genomic regions implicated in phenotypic variability between breeds [68,69]. SB breeds are completely black (skin and hair), although there are some animals that present gray hair on the body [69]. D’Alessandro et al. performed a genome-wide analysis to identify regions that explain the phenotypic differences between Nero Siciliano and Gray Siciliano individuals [69]. In this study, using different approaches, two significant regions, SSC5 and SSC15, were identified, and they contained different candidate genes linked to growth traits in pigs. In particular, a marker gene near MGAT4C, a gene associated with ADG in pigs, was identified. Furthermore, the two groups showed different runs of homozygosity islands, with several candidate genes involved in coat color (in Sicilian Gray) or related to different pig performance traits (in Nero Siciliano). In this population of SB, the “Faccioli pig” [3] and other white patterns that may appear on extreme portions of legs could appear in this population. Schiavo et al. [70] conducted a GWAS to identify genomic regions that can affect coat color variability in SB pigs. A significant marker on SSC2 was associated with white coat patterns in this breed. This polymorphism was placed in the QTL region for black coats by Hirooka et al. [71]. Another study was focused on analyzing candidate genes for meat quality between SB and Italian heavy pig genomes. In particular, the authors focused on genes related to muscle mass deposition and carcass fatness, as these traits influence technological processes adopted for long matured pork meat products such as cured ham. More than 20,000 variants have been identified by comparing the gene set of each breed with the assembly of the reference genome. Of these, ~22,000 were SNPs, ~3000 were short insertions, and ~1400 short deletions. The transition/transversion ratio was 2650, while the missense/silent ratio was 0.526. Of all variants detected in this study, more than 3000 were shared by Nero Siciliano, Large White, and Landrace, while ~7000 were unique for SB, ~2000 for Large White, and ~6000 for Landrance, showing a high degree of genetic variability among the breeds studied [72].

## 10. Conclusions

This manuscript aims to provide an overview of the Sicilian Black (SB) pig, offering useful information on phenotypic, productive, reproductive, and genetic characteristics. These aspects can play an important role in the development of new genetic selection concepts, useful for creating innovative sustainable chains based on autochthonous pig breeds. In the Italian territory, especially in Sicily, animal genetic resources and their rearing systems are an integral part of ecosystems and productive landscapes. Indeed, this overview summarizes the reasons for the conservation of genetic diversity in autochthonous pig populations that may be related to biological, social, cultural, and economic aspects. Traditional production systems required multipurpose animals, which, although less productive than high-yielding breeds, may contain valuable functional traits. Although local breeds are usually not competitive for production traits, they can have valuable characteristics, such as disease resistance and distinctive product quality. The greater economic value of typical productions compared to conventional commercial products and the growing preferences of consumers towards food quality could strongly support and direct the conservation plan of zootechnical biodiversity. Innovative methods based on molecular genetics are playing a relevant role in providing effective tools for the conservation of autochthonous genetic resources, and in monitoring and safeguarding their genetic variability, thus contributing to the enhancement of traditional food production systems that show high nutritional and organoleptic values. As genomic information continues to provide extremely valuable biological information, the key to further genomic selection and successful genomic approaches will be to collect the most relevant phenotypes, identify causal mutations and the exact mechanisms by which the best phenotypes are produced.

## Figures and Tables

**Table 1:** Summary of morphology information.

Morphological Traits ^1^	Adult Male	Adult Female
Body weight (kg)	148	128
Body length (cm)	104	90
Ear length	small	small
Chest girth (cm)	131	120
Height at withers (cm)	62–67	62–67
Number of teat	11.4	11.4

^1^ ANAS, 2013—Italian Pig Breeders Association. Available online: http://www.anas.it/Normative/Norme001 (accessed on 18 July 2020).

**Table 2:** Overview on growth performance in ad libitum and restrictive feeding.

Feeding	ADG Fattening ^1^			ADG Birth-Slaughter	Ref.
	Early	Middle	Overall		
-	-	-	600	-	[1]
-	-	-	-	211	[30]
-	-	-	253	-	[31]
-	-	-	191	-	[31]
Restrictive	-	264	264	-	[33]
Restrictive	-	162	162	-	[33]
Restrictive	-	-	431	-	[33]
Ad libitum	-	-	540	-	[33]
Restrictive	328	-	328	-	[34]
Restrictive	360	-	360	-	[35]
-	241	333	287	-	[37]
Restrictive	-	-	208	-	[38]
-	-	465	465	-	[39]
-	-	346	346	-	[40]
Restrictive	-	-	358	-	[40]
Restrictive	-	-	393	-	[40]

ADG = average daily gain in g; ^1^ ADG in a period of fattening is reported for early and middle fattening stages estimated between 30 and 60 kg and 60 and 100 kg, respectively.

**Table 3:** Overview on body composition and carcass traits.

Final Age (d)	Final Body Weight (kg)	Hot Carcass Weight (kg)	Dressing Yield (%)	Lean Meat Content (%)	BFT (mm)			Ref.
					S ^1^	At Withers	At Last Rib	
-	-	105	80.0	-	-	-	-	[1]
380	96	78	81.1	-	-	-	-	[31,32,33]
452	86	71	82.9	-	-	-	-	
-	121	98	81.0	-	-	-	42	[41,42]
-	110	88	80.5	-	-	-	34	
-	97	77	79.4	58.2	-	-	42	
-	102	82	80.8	59.0	-	-	49	
-	110	89	80.6	-	40	-	34	[34]
-	100	81	81.2	-	30	-	28	
169	62	45	78.7	48.7	-	-	17	[34,35,36]
169	67	54	79.9	49.9	-	-	23	
339	83	64	76.8	42.3	35	-	32	[32,33]
339	93	74	79.1	39.7	49	-	39	
448	102	83	82.5	-	45	-	37	[37,38]
487	88	82	81.9	-	39	-	33	
730	107	89	82.9	-	46	52	47	[39]

BFT = back fat thickness (mm); ^1^ S: back fat thickness (BFT) measured according to Fat-o-Meter (FOM method) method.

**Table 4:** Overview on Sicilian black pig meat quality.

pH 45	pH 24	CIE ^1^			IMF (%)	IMF Fatty Acid Composition ^2^				BFT Fatty Acid Composition ^3^				Ref.
		L*	a*	b*		SFA	MUFA	PUFA	n6/n3	SFA	MUFA	PUFA	n6/n3	
6.07	5.51	52	-	-	-	-	-	-	-	-	-	-	-	[32]
-	-	49	-	-	-	-	-	-	-	-	-	-	-	
6.12	-	49	10.6	10.1	3.7	39.66	48.9	11.44	3.4	-	-	-	-	[41]
6.28	-	46	10.1	11.4	3.0	34.04	59.93	6.03	13.0	-	-	-	-	
6.38	-	-	-	-	10.0	-	-	-	-	-	-	-	-	
6.14	-	-	-	-	5.7	-	-	-	-	-	-	-	-	
6.28	5.65	46	11.0	12.4	5.6	35.7	58.9	5.44	37.8	-	-	-	-	[34]
6.38	5.64	47	11.4	13.4	4.6	35.6	58.6	5.79	33.1	-	-	-	-	
6.37	5.65	61	-	-	2.7	38.6	49.3	12.1	7.5	40.9	49.1	10.0	9.7	[35,36,44,45]
6.34	5.56	61	-	-	3.1	41.4	49.5	9.1	10.8	41.0	47.6	11.4	13.8	
6.29	-	47	15.3	4.9	3.3	-	-	-	-	38.3	47.4	14.5	18.3	[38,39,44,45]
6.18	-	50	14.7	5.8	4.3	-	-	-	-	35.8	53.3	10.9	10.7	
6.06	5.45	51	15.7	4.6	-	-	-	-	-	-	-	-	-	[40]

pH 45 = pH was measured approximately 45 min post-mortem; pH 24 = pH was measured approximately 24 h post-mortem; IMF = intramuscular fat; SFA = saturated fatty acids; MUFA = monounsaturated fatty acids; PUFA = polyunsaturated fatty acids. ^1^ CIE = Objective colour defined by Commission Internationale de l’Eclairage; L* greater value indicates lighter colour; a* greater value indicates a redder colour; b* greater value indicates a more yellow colour. ^2^ For fatty acid composition of intramuscular fat tissue in longissimus muscle, only pigs on control diet were considered, and when fatty acid composition was reported separately for neutral and polar lipids, values reported for neutral lipids were considered. Control diets differed among studies—to see diet composition refer to the corresponding source. ^3^ For fatty acid composition of back fat tissue, only pigs on control diet were considered and when fatty acid composition was reported separately for outer and inner layers, values reported for outer layer of back fat tissue were considered. Control diets differed among studies—to see diet composition refer to the corresponding source.
